# A high-throughput LC-MS/MS method for the measurement of the bile acid/salt content in microbiome-derived sample sets

**DOI:** 10.1016/j.mex.2020.100951

**Published:** 2020-06-12

**Authors:** Thomas D. Horvath, Sigmund J. Haidacher, Kathleen M. Hoch, Jennifer M. Auchtung, Anthony M. Haag

**Affiliations:** a Department of Pathology and Immunology, Baylor College of Medicine, 1 Baylor Plaza, Houston, TX 77030, United States; b Texas Children's Microbiome Center, Texas Children's Hospital, 1102 Bates Ave, Houston, TX 77030, United States; c Food Science and Technology Department, the University of Nebraska-Lincoln, 1400 R Street, Lincoln, NE 68588, United States

**Keywords:** Bioanalytical chemistry, High-throughput sample analysis, High-performance liquid chromatography, LC-MS/MS, Microbiome, Microbiology

## Abstract

Due to the physicochemical properties of bile acids/salts (*i.e*., hydrophobic and ionizable), the application of reverse-phase liquid chromatography-tandem mass spectrometry (LC-MS/MS)-based methods are ideally suited for the measurement of these compounds in a host of microbiologically-relevant matrices. Here, we provide a detailed bioanalytical protocol that contains several modifications of a method previously described by Wegner et al. [1]. Briefly, this modified method exhibits the following advantages for the measurement of cholic acid (CA), taurocholic acid (TCA), and deoxycholic acid (DCA) in microbiome-relevant sample matrices: i) fecal sample processing has been streamlined by the elimination of lyophilization and manual homogenization steps; ii) the Sciex 6500 QTRAP hybrid triple-quadrupole/linear ion trap mass spectrometer has sufficient sensitivity to perform the measurement of bile acids/salts in negative ion mode – ammonium adducts of bile acids/salts are not required for detection; and, iii) assay throughput has been boosted by more than 5-fold by shortening the chromatographic duty cycle of a single sample injection from 45 min to 8.4 min. Recently, the method was used to perform 508 sequential injections (72 calibration standards, 52 blank-internal standard sample, and 368 MiniBioReactor Array (MBRA)-derived samples) from four separate batches over a 4-day time period.

## Graphical abstract

**Figure:**
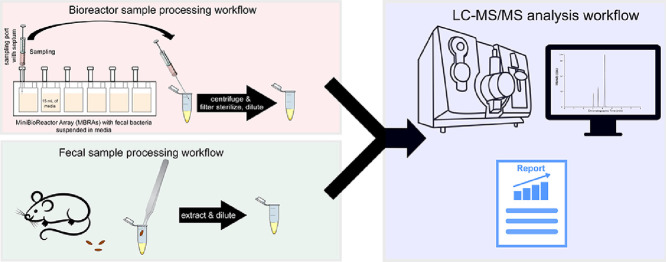

Specifications Table

**Table:** 

Subject Area:	Immunology and Microbiology
More specific subject area:	Microbiome-related research projects
Protocol name:	Measurement of bile acids/salts in microbiome relevant matrices by LC-MS/MS
Reagents/tools:	Materials Optima LC/MS-grade water, methanol, and acetonitrile, and ACS reagent-grade, absolute ethanol (200 proof) were from Thermo Fisher Scientific (Waltham, MA, USA). Ammonium formate (99%), phosphate buffered saline (PBS), and dimethyl sulfoxide (DMSO) were from Millipore Sigma (Billerica, MA, USA). The authentic reference materials for sodium cholic acid (CA) and sodium deoxycholic acid (DCA) were from Thermo Fisher Scientific, and sodium taurocholic acid (TCA) was from Millipore Sigma. Authentic D_4_-cholic acid (D_4_—CA) and D_4_-deoxycholic acid (D_4_-DCA) Internal Standard (IS) reference materials were purchased from CDN Isotopes (Pointe-Claire, Quebec, Canada). Polyvinyl difluoride (PVDF) membrane filter plates with 0.2 μm pores were from Thermo Fisher Scientific. High-Performance Liquid Chromatography (HPLC) separations were performed using a Raptor C18 (2.7 µm superficially porous silica particle (SPP), 100 mm (L) x 2.1 mm (ID), 90 Å pore size; Cat #9304A12) analytical column and an Ultra C18 (5.0 µm silica, 10 mm (L) x 2.1 mm (ID), 100 Å pore size; Cat #917450212) guard column from Restek (Bellefonte, PA, USA). MiniBioReactor Array (MBRA) devices were constructed and used as described previously [11].
	Biological Materials The custom bioreactor medium used for this study has been described previously [11]. The protocols for the harvesting and processing of bacterial cells from human fecal communities used for this study were described previously [11]. The protocols for the collection and processing of mouse fecal pellets used for this study were described previously [12]. HPLC-tandem mass spectrometry (LC-MS/MS) System The method was developed on an LC-MS/MS system comprised of the following components: i) a Genius 3031 nitrogen generator by Peak Scientific (Inchinnan, Scotland, UK); ii) a Security Plus uninterruptable power supply by Powervar (Tustin, CA, USA); iii) a Nexera X2 MP HPLC system by Shimadzu (Kyoto, Japan); iv) a Sciex 6500 QTRAP hybrid triple-quadrupole/linear ion trap mass spectrometer by Danaher (Washington, DC, USA); and, v) a Dell Optiplex XE2 personal computer that has been configured for LC-MS/MS instrument control.
Experimental design:	This method provides a detailed description of the materials, equipment, and methods necessary for performing LC-MS/MS-based quantitative bioanalysis of the CA, TCA, and DCA content of sample matrices pertinent to understanding host-microbiome interactions.
Trial registration:	N/A
Ethics:	N/A
Value of the Protocol:	Advantages of this method include: 1) Bile acids/salts are multifunctional compounds of great interest to the microbiome-based research community. 2) LC-MS/MS-based methods can measure the bile acid/bile salt content in bioreactor-derived broth media and stool samples 3) The method can be considered high-throughput – we are able to process up to 192 MiniBioReactor Array (MBRA) or mouse fecal samples per 8 h day, and can perform the instrumental analysis of these samples in a 36 h timeframe.

*Description of protocol:*[This protocol contains refined extraction, chromatographic, and MS/MS procedures that enhance the analytical throughput of an existing quantitative LC-MS/MS-based bile acid/salt method**[1]*. *The method modifications described herein were necessary to accommodate the large sample counts associated with ongoing microbiome projects supported by the Texas Children’s Microbiome Center--Mass Spectrometry Laboratory (TCMC-MSL). The procedural modifications yielded the following method improvements: i) the acquisition duty cycle was reduced from 45 min to 8.4 mins per sample; ii) the processing rate was increased to 192 samples per 8 h day; and, iii) chromatographic data for the deprotonated precursor ions of the bile acids/salts were measured directly using the negative ionization mode of a Sciex 6500 QTRAP MS system, rather than measuring the ammoniated adducts of bile acids/salts in positive ionization mode as previously reported**[1]*.*]*

## Biochemical background

**Fig. 1:**
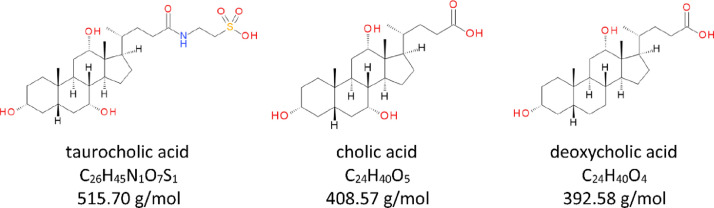
Molecular structure, chemical formula, and molar mass for host-derived taurocholic acid (TCA), and microbiota-derived cholic acid (CA) and deoxycholic acid (DCA) contained in fecal or bioreactor-derived broth media samples.[7]

In the human liver, primary bile acids chenodeoxycholic acid (CDCA) and cholic acid (CA) are formed by catabolism of cholesterol [2,3], and are conjugated to glycine and taurine to form the hydrophilic bile salts glycocholic acid (GCA) and glycochenodeoxycholic acid (GCDCA), and taurocholic acid (TCA) and taurochenodeoxycholic acid (TCDCA), respectively (Figure 1)[4,5]. After formation, bile salts enter the enterohepatic cycle where they are: i) exported with other hydrophobic components to form bile in the gall bladder [6]; ii) secreted into the duodenum where they aid in the absorption and digestion of hydrophobic dietary components; and, iii) are reabsorbed (~95% of the pool) from the chyme in the distal ileum and are transported back to the liver via the portal vein thus completing a single revolution of the enterohepatic cycle [6]. In the human gut, a small proportion (~5%) of the bile salt pool undergoes microbiota-mediated deconjugation of glycine and taurine to reform the hydrophobic, cytotoxic, and potentially antimicrobial primary bile acids CDCA and CA [[7], [8]–9], and these compounds may be metabolized further to the secondary bile acids, deoxycholic acid (DCA) and lithocholic acid (LCA) by microbiota in the large bowel [10].

## Solution preparations

### Internal standard (IS) solution preparations

1. Individual Internal Standard (IS) Stock Solutions for D_4_-cholic acid (D_4_—CA) and D_4_-deoxycholic acid (D_4_-DCA) are each prepared at a concentration of ~10 mg/mL in Optima LC/MS-grade water (grade of water used throughout the protocol), and in a solution of 1:2 dimethyl sulfoxide: water, respectively. These solutions are stored frozen at −80 °C while not in use. *Note: Free D_4_-DCA is relatively water insoluble, therefore it may be necessary to use a combination of vortex-mixing (at 2000 RPM for 5 min) and gentle heating (at 70* °*C for 1* min*) in a block heater to re-solubilize the bile acid/salt content in the freshly thawed stock solution preparations.*
2. A Combined IS Intermediate Solution is prepared by diluting appropriate volumes of the D_4_—CA and D_4_-DCA IS Stock Solutions in an appropriate volume of 1:1 methanol: water solution to yield a concentration of 500 ng/mL for each compound in the combined solution. This solution is refrigerated at +4 °C while not in use.
3. A Working IS-A (WIS-A) Solution is prepared by diluting a volume of Combined IS Intermediate Solution in an equal volume of 1:1 methanol: water solution yielding a concentration of 250 ng/mL for each IS component (D_4_—CA and D_4_-DCA). This solution is refrigerated at +4 °C while not in use, and is discarded after 1 week of storage. *Note: This solution is used as the diluent for all microbiome samples prior to analysis.*
4. A Working IS-B (WIS-B) Solution is prepared by diluting a 9 mL volume of the WIS-A Solution with a 1 mL volume of 1:1 methanol: water solution yielding a concentration of 225 ng/mL for each IS component (D_4_—CA and D_4_-DCA). This solution is refrigerated at +4 °C while not in use, and is discarded after 1 week of storage. *Note: This solution is used as the diluent in the preparation of the CA, DCA, and TCA containing Working Analyte Solution, and the serially-diluted LC-MS/MS calibration standards.*

### Analyte solution preparations

1. Individual Analyte Stock Solutions for CA, DCA, and TCA are each prepared at a concentration of ~100 mg/mL in water. These solutions are stored frozen at −80 °C while not in use. *Note: sodium salts of bile acids are readily water soluble, but it may still be necessary to re-solubilize the bile acid/salt content in the stock solutions. A combination of vortex-mixing (at 2000 RPM for 5 min) and gentle heating (at 70* °*C for 1* min*) may be used to re-solubilize insoluble bile acid/salt material.*
2. A Combined Analyte Intermediate Solution is prepared by diluting appropriate volumes of the CA, DCA, and TCA Analyte Stock Solutions in an appropriate volume of water to yield a concentration of 10.0 mg/mL for each compound in the combined solution. This solution is sub-aliquotted at 100 µL volumes and stored frozen at −80 °C while not in use.
3 A Working Analyte Solution is prepared by diluting an appropriate volume of the Combined Analyte Intermediate Solution in an appropriate volume of the WIS-B Solution to yield a concentration of 100 µg/mL for each analyte component (CA, DCA, and TCA). This solution is refrigerated at +4 °C while not in use, and is discarded after 1 week of storage.
4. The LC-MS/MS calibration standards (Calibrators) are prepared by serial dilution using the Working Analyte Solution as the primary source solution and the WIS-B solution as the diluent (See Table 1). These Calibrators are refrigerated at +4 °C while not in use, and are discarded after 1 week of storage.

**Table 1:** Serial dilution scheme for the preparation of the bile acid/salt Calibrators.

Calibrator	CA, DCA, and TCA concentration in each Calibrator (ng/mL)	Source Solution	Volume of Source Solution (µL)	Volume of Working IS-B Solution (µL)
F	1000	Working Analyte Solution	10	990
E	250	F	100	300
D	62.5	E	100	300
C	15.6	D	100	300
B	3.90	C	100	300
A	0.977	B	100	300

## Microbiome sample preparation, shipment, and storage and handling conditions

### Bioreactor sample collection, filter-sterilization, and extraction procedures

Bacterial cells from human fecal communities are grown in the bioreactor medium contained in the MBRA devices [11]. Bioreactor medium contains bovine bile as a source of bile salts. Samples are removed from communities and cells are pelleted by centrifugation at 3000 *g* for 5 min. Clarified supernatants are filtered through 0.2 μm polyvinyl difluoride (PVDF) membrane filter plates by centrifugation at 200 *g* for 5 min. Filtered samples are stored frozen at −80 °C while awaiting shipment to the Texas Children's Microbiome Center Mass Spectrometry Laboratory (TCMC-MSL).

### Mouse stool collection, filter-sterilization, and extraction procedures

Fecal samples are collected from human microbiota associated mice and stored at −80 °C [12]. Fecal pellets (30–60 mg) are fully resuspended in a 250 μL volume of phosphate buffered saline (PBS), and are centrifuged at 20,000 *g* for 1 min. The clarified supernatant is transferred to a new tube. Decanted fecal material is resuspended in a 250 μL volume of absolute ethanol (200 proof). The ethanolic samples are centrifuged at 20,000 *g* for 1 min. Equal volumes of the clarified ethanolic and PBS supernatants are combined and filtered through 0.2 μM PVDF membrane filter plates by centrifugation at 200 *g* for 5 min. Filtered samples are stored frozen at −80 °C while awaiting shipment to the TCMC-MSL.

### Long-term sample storage and handling

1. Samples (bioreactor media or stool extracts) that are generated offsite are shipped to the TCMC-MSL on dry ice, and upon receipt are transferred immediately into a −80 °C freezer for storage until further processing and analysis may be performed. Samples that are generated in the TCMC are filter-sterilized if needed, and are then transferred to a −80 °C freezer as soon as possible.
2. All microbiome-derived samples are thawed at ambient temperature on the laboratory benchtop.
3. If samples are provided in 96-well plates, then the entire sample volume contained in each well is transferred to individual 0.6 or 1.5 mL Eppendorf tubes, and processed further according to the following protocols. All samples are vortex-mixed for 2 min using a multi-tube vortexer. Stool sample extracts are centrifuged at 10,000 *g* for 10 min to settle the debris prior to sample aliquoting and dilution. After sample processing has been completed for the entire batch, the residual sample volumes contained in the Eppendorf tubes are stored frozen at −80 °C pending re-analysis.

## Sample dilution procedures

### 1000-fold sample dilutions for MBRA-derived bioreactor media samples

1. For this particular study, it was determined that a 1000-fold dilution was suitable for the levels of CA and DCA contained in the samples. Dilution factor suitability should be assessed for each new study prior to sample analysis.
2. A 100-fold primary dilution is performed by mixing a 10 µL volume of the undiluted bioreactor sample in a 990 µL volume of a 1:1 methanol: water solution. The diluted samples are then vortex-mixed for 15 s.
3. A secondary sample dilution is performed directly in a polypropylene autosampler injection vial by mixing a 10 µL volume of the 100-fold diluted bioreactor sample in 90 µL of the WIS-A Solution for an overall dilution factor of 1000-fold. The diluted sample is then vortex-mixed for 15 s, and a 5 µL volume of sample is injected onto the LC-MS/MS system. *Note: The 10% dilution of the WIS-A solution during sample preparation is accounted for in the preparation of the WIS-B IS solution that is used in the preparation of the calibrators.*

### 10-fold sample dilutions for stool (CA, DCA, and TCA) and MBRA-derived bioreactor (TCA only) samples

1. For this study, it was determined that a 10-fold dilution was suitable for the levels of TCA contained in the MBRA-derived bioreactor samples, and for the CA, DCA, and TCA contained in the mouse fecal samples. Dilution factor suitability should be assessed for each new study prior to sample analysis.
2. A 10-fold dilution is performed directly in a polypropylene autosampler injection vial by mixing a 10 µL volume of the undiluted bioreactor/stool extract sample in 90 µL of the WIS-A Solution. The diluted sample is then vortex-mixed for 15 s, and a 5 µL volume of sample is injected onto the LC-MS/MS system. *Note: The 10% dilution of the WIS-A solution during sample preparation is accounted for in the preparation of the WIS-B IS solution that is used in the preparation of the calibrators.*

## LC-MS/MS method details

### Mobile phase and needlewash solution preparations

1. The Mobile Phase A (MPA) Solution is prepared by dissolving 0.631 g of ammonium formate in 1 L of water in a 1 L HPLC solvent reservoir bottle. The MPA Solution is swirled vigorously to ensure complete dissolution of the salt, and is stored well-closed at ambient room temperature while not in use, and the solution is discarded after 1 month.
2. The Mobile Phase B (MPB) Solvent is pure Optima LC/MS-grade acetonitrile and is stored well-closed at ambient room temperature while not in use. The pure solvent does not expire.
3. The Needlewash (NW) Solution is prepared by mixing equal parts of water and Optima LC/MS-grade methanol in an HPLC solvent reservoir bottle. The NW Solution swirled vigorously to ensure complete mixing, is degassed via sonication, and is stored well-closed at ambient room temperature while not in use. This solution does not expire.

### Chromatographic method

**Fig. 2:**
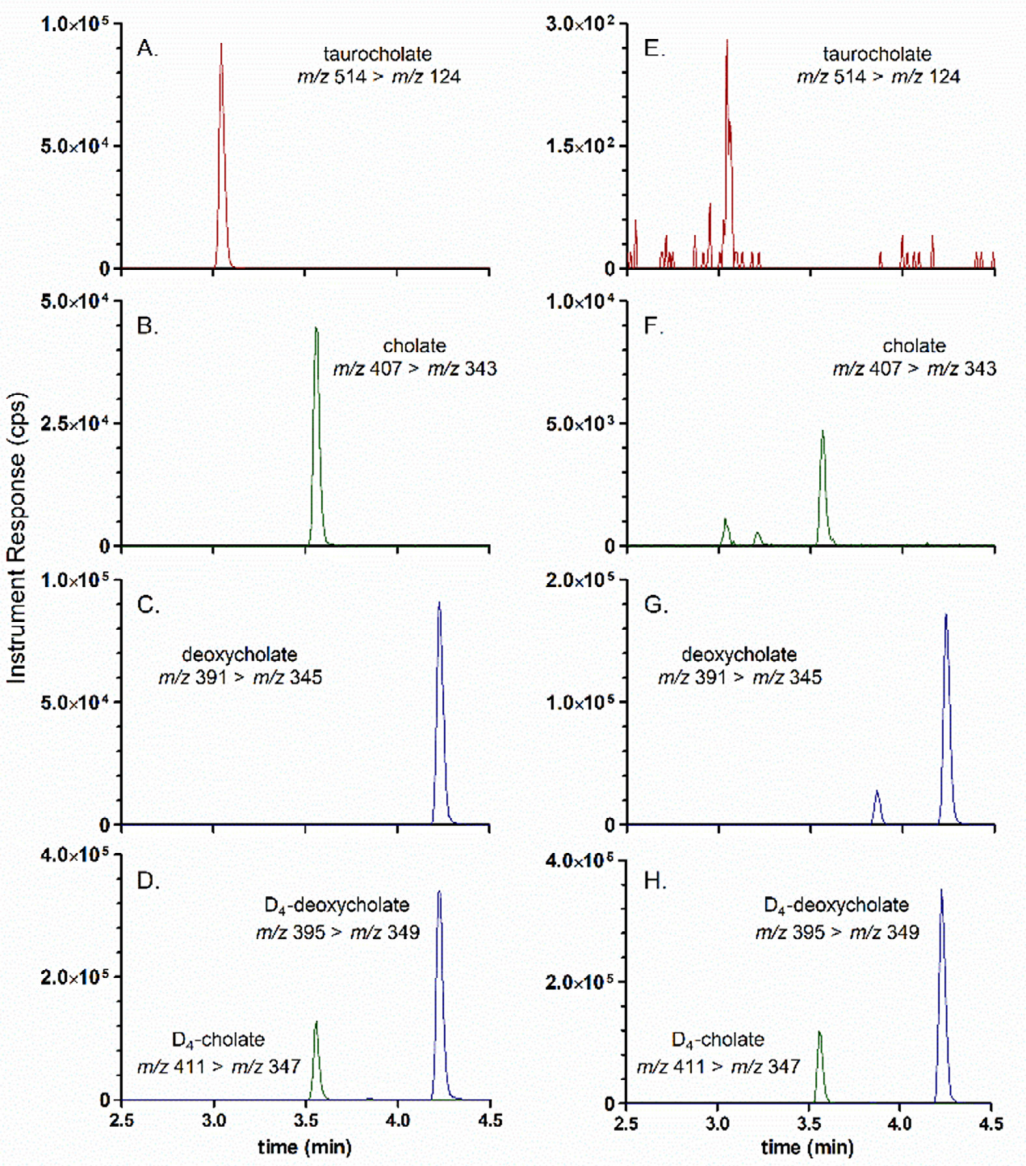
(A-D) Extracted-Ion Chromatograms (XICs) for a calibration standard that contained 62.5 ng/mL of taurocholate (A), cholate (B), deoxycholate (C), and 225 ng/mL for the internal standards D_4_-cholate (D; green trace) and D_4_-deoxycholate (D; blue trace). (E-H) XIC chromatograms that illustrate the instrument Responses (counts per second (CPS) for taurocholate (E), cholate (F), deoxycholate (G), and the internal standards (H) for a 1000-fold diluted bioreactor media sample.

1. Reverse-phase chromatography is performed using a Raptor C18 (2.7 µm SPP particles, 100 mm (L) x 2.1 mm (ID), 90 Å pore size; Cat #9304A12) analytical column with an Ultra C18 (5.0 µm silica, 10 mm (L) x 2.1 mm (ID) mm, 100 Å pore size; Cat #917450212) guard column installed.
2. The needle rinse program on the Nexera X2 MP Autosampler is specified to perform external rinsing only both before and after aspiration of sample using 500 µL of NW Solution each time.
3. The chromatographic method includes a column temperature of 50 °C, an autosampler tray temperature of 15 °C, a mobile phase flowrate of 0.30 mL/min, and a gradient elution program specified as follows: 0–4.50 min, 5–90% MPB; 4.50–5.50 min, 90% MPB; 5.50–5.60 min, 90%−5% MPB; 5.60–8.00 min, 5% MPB; with an overall cycle time of 8.4 min per acquisition, and an operational back pressure of ~2200 psi at initial conditions. The 6500 QTRAP divert valve was enabled and specified to divert the chromatographic eluate according to the following program: 0–1.00 min, divert to waste (position B); 1.00–8.00 min, divert to MS source (position A); 8:00–8.40 min, divert to waste (Position B).
4. The retention times have typically been 3.1 min for TCA, 3.6 min for CA, and 4.25 min for DCA (Fig. 2).

## Column efficiency characterization

The column efficiency characterization for the Raptor C18 analytical and Ultra C18 guard column combination was performed by computing the pertinent experimental parameters from a mid-level calibration standard (Calibrator D – prepared at 62.5 ng/mL for CA, DCA, and TCA, and 225 ng/mL for the IS compounds, D_4_—CA and D_4_-DCA) using previously described methods [13]. Briefly, the height equivalent to a theoretical plate (HETP; Eq (1)) and the number of theoretical plates per meter (N/m; Eq (2)) were calculated using the following experimental parameters for each analyte and IS compound: i) full-width at ½ max for the chromatographic peaks (*W_1/2_*) in minutes; ii) the chromatographic retention time (τ_R_) in minutes; and, iii) the column length (L) specified in the units of cm for the computation of HETP (Eq. (1)), and in the units of m for N/m (Eq. (2)). All experimental parameters used to perform the column efficiency characterization have been tabulated in Table 2 below.

(1) $$HETP\;\left(\frac{cm}{plate}\right)=\;\frac{{\left(W_{1/2}\right)}^{2}*L\;\left(cm\right)}{5.54*\;{\left(\tau_{R}\right)}^{2}}$$

(2) $$plates\;per\;meter\;\left(\frac{N}{m}\right)=\;\frac{5.54\;*\;{\left(\tau_{R}\right)}^{2}}{{\left(W_{1/2}\right)}^{2}*\;L\;\left(m\right)\;}$$

**Table 2:** Column efficiency characterization using a mid-level calibrator prepared at 62.5 ng/mL for all analytes and 250 ng/mL for all IS compounds.

Analyte / IS	MS/MS transition	Column Length (L; cm)	Retention Time (τ_R;_ min)	Full width at half-max (W_1/2_; mins)	HETP (cm/plate)	Plates per meter (N/m)
CA	407 > 343	10	3.56	0.035	0.00017	590,000
D_4_—CA (IS)	411 > 347	10	3.56	0.031	0.00014	720,000
DCA	391 > 345	10	4.23	0.040	0.00016	640,000
D_4_-DCA (IS)	395 > 349	10	4.22	0.039	0.00015	670,000
TCA	514 > 124	10	3.04	0.031	0.00019	520,000

### MS/MS acquisition method

1. The TurboIonSpray^Ⓡ^ electrospray ionization (ESI) probe is installed in the Turbo V™ ion source and is operated with the following source conditions: ionization mode polarity: negative; curtain gas (Cur): 20; TurboIonSpray™ voltage (IS): −4500 V; source temperature (TEM): 250 °C; Ion Source Gas 1 (GS1; nebulization gas): 40; Ion Source Gas 2 (GS2; heater gas): 50.
2. MS operational parameters include the following: collisionally activated dissociation (CAD) gas pressure: “High”; declustering potential (DP): −40 V; entrance potential: −8 V; collision-cell exit potential (CXP): −10 V; mass analyzer quadrupole 1 (Q1) resolution setting: unit; Q3 resolution: unit; molecule specific parameter for each bile acid analyte are tabulated in Table 3.

**Table 3:** The molecule-specific MS parameters for cholic acid, deoxycholic acid, and taurocholic acid.

Q1 (*m/z*)	Q3 (*m/z*)	Dwell Time (mS)	ID	Collision energy (CE) (V)
407.3	343.3	50	Cholic acid 407 > 343	−47
407.3	289.3	50	Cholic acid 407 > 289	−52
411.3	347.3	50	Cholic acid-D4 411 > 347	−47
411.3	290.3	50	Cholic acid-D4 411 > 290	−52
391.3	345.3	50	Deoxycholic acid 391 > 345	−47
391.3	355.3	50	Deoxycholic acid 391 > 355	−46
395.3	349.3	50	Deoxycholic acid-D4 395 > 349	−47
395.3	359.3	50	Deoxycholic acid-D4 395 > 359	−46
514.3	124.1	50	Taurocholic acid 514>124	−64
514.3	107.0	50	Taurocholic acid 514 > 107	−64

## Method performance characteristics

A total of four analytical batches were prepared across two separate days – each batch consisted of a calibration curve, 13 interspersed blanks, and 96 MBRA-derived media samples that had been diluted according to the procedures described above. Calibration curves were constructed for each analyte by plotting the instrument response (IR = A_analyte_ / A_IS_) factor of each Calibrator against their respective nominal concentration. From this plot, a least-squares, linear regression with weighting (1/x) was used to calculate the line of best fit for each analyte, and yielded the following representative calibration curves for each analyte: CA: IR_CA_ = 0.00526*[CA] + 0.00651, R^2^ = 0.991; TCA: IR_TCA_ = 2310*[TCA] +3045, R^2^ = 0.9856; DCA: IR_DCA_ = 0.00337*[DCA] + 0.00641, R^2^ = 0.999. Limit of Detection (LOD) and Limit of Quantitation (LOQ) estimates were calculated using the standard deviation of the y-intercepts and the mean slope of the four calibration curves calculated for each analyte as described previously [14]. The LOD and LOQ estimates for each analyte are tabulated with the mean linear regression parameters for the four calibration curves (± Standard Error of the Mean (SEM)) in Table 4.

**Table 4:** Mean weighted (1/x) linear regression parameters for cholic acid (CA), taurocholic acid (TCA), and deoxycholic acid (DCA) calibration curves (*n* = 4) over a dynamic range of 0.977 to 1000 ng/mL.

Analyte	Internal Standard (IS)	LOD / LOQ (ng/mL)	Slope (mean ± SEM)	Y-intercept (mean ± SEM)	R^2^ (mean ± SEM)
CA	D_4_—CA	0.20 / 0.63	0.00562 ± 1.8e^−4^	0.00600 ± 1.8e^−4^	0.999 ± 1.8e^−4^
TCA*	–	0.76 / 2.3	2410 ± 116	2800 ± 280	0.989 ± 1.3e^−3^
DCA	D_4_-DCA	1.1 / 3.4	0.00359 ± 1.2e^−4^	0.00359 ± 1.2e^−4^	0.999 ± 1.0e^−4^

⁎ A commercial source of D_4_-TCA Internal Standard reference material was not found during development.

## Discussion

Herein, we report on a high-throughput LC-MS/MS-based bioanalytical method that is suitable for the quantitation of CA, TCA, and DCA in microbiome relevant matrices such as stool and filter-sterilized MBRA-derived bioreactor broth media. Using this method, TCMC-MSL staff are capable of processing up to 192 stool or bioreactor samples in a single 8 h day, and the LC-MS/MS acquisition of all calibrators, blanks, and unknown samples will require ~36 h to complete for the two analytical batches. This method is highly adaptable, and with the purchase of authentic reference standards, can be modified to include other bile acids/salts that may be of interest TCMC-MSL collaborators.

## Credit author statement

Jennifer Auchtung, Anthony Haag: Conceptualization; Anthony Haag, Sigmund Haidacher: Methodology; Thomas Horvath, Sigmund Haidacher: Validation and Investigation; Kathy Hoch, Thomas Horvath: Formal Analysis and Data curation; Thomas Horvath: Writing- Original draft preparation and Visualization; Anthony Haag: Supervision; Thomas Horvath, Jennifer Auchtung, and Anthony Haag: Writing- Reviewing and Editing; Jennifer Auchtung, Anthony Haag: Funding acquisition.

## Declaration of Competing Interest

The authors declare no conflicts of interest
